# A double blind randomised controlled trial comparing standard dose of iron supplementation for pregnant women with two screen-and-treat approaches using hepcidin as a biomarker for ready and safe to receive iron

**DOI:** 10.1186/s12884-016-0934-8

**Published:** 2016-07-13

**Authors:** Amat Bah, Rita Wegmuller, Carla Cerami, Lindsay Kendall, Sant-Rayn Pasricha, Sophie E. Moore, Andrew M. Prentice

**Affiliations:** MRC Unit The Gambia & MRC International Nutrition Group, PO Box 273, Banjul, The Gambia; MRC Unit The Gambia, PO Box 273, Banjul, The Gambia; The Weatherall Institute of Molecular Medicine, University of Oxford, John Radcliffe Hospital, Oxford, OX3 9DS UK; MRC Human Nutrition Research, Elsie Widdowson Laboratory, 120 Fulbourn Road, Cambridge, CB1 9NL UK

**Keywords:** Pregnancy, Hepcidin, Anaemia, UNIMMAP, Lower dose iron, Iron deficiency

## Abstract

**Background:**

Until recently, WHO recommended daily iron supplementation for all pregnant women (60 mg/d iron combined with 400ug/d folic acid) where anaemia rates exceeded 40 %. Recent studies indicate that this may pose a risk to pregnant women. Therefore, there is a need to explore screen-and-treat options to minimise iron exposure during pregnancy using an overall lower dosage of iron that would achieve equivalent results as being currently recommended by the WHO. However, there is a lack of agreement on how to best assess iron deficiency when infections are prevalent. Here, we test the use of hepcidin a peptide hormone and key regulator of iron metabolism, as a potential index for ‘safe and ready to receive’ iron.

**Design/Methods:**

This is a 3-arm randomised-controlled proof-of-concept trial. We will test the hypothesis that a screen-and-treat approach to iron supplementation using a pre-determined hepcidin cut-off value of <2.5 ng/ml will achieve similar efficacy in preventing iron deficiency and anaemia at a lower iron dose and hence will improve safety. A sample of 462 pregnant women in rural Gambia will be randomly assigned to receive: a) UNU/UNICEF/WHO international multiple micronutrient preparation (UNIMMAP) containing 60 mg/d iron (reference arm); b) UNIMMAP containing 60 mg/d iron but based on a weekly hepcidin screening indicating if iron can be given for the next 7 days or not; c) or UNIMMAP containing 30 mg/d iron as in (b) for 12 weeks in rural Gambia. The study will test if the screen-and-treat approach is non-inferior to the reference arm using the primary endpoint of haemoglobin levels at a non-inferiority margin of 0.5 g/dl. Secondary outcomes of adverse effects, compliance and the impact of iron supplementation on susceptibility to infections will also be assessed.

**Discussion:**

This trial is expected to contribute towards minimising the exposure of pregnant women to iron that may not be needed and therefore potentially harmful. If the evidence in this study shows that the overall lower dosage of iron is non-inferior to 60 mg/day iron, this may help decrease side-effects, improve compliance and increase safety. The potential for the use of hepcidin for a simple point-of-care (PoC) diagnostic for when it is most safe and effective to give iron may improve maternal health outcomes.

**Trial registration:**

ISRCTN21955180

## Background

Anaemia is a global public health problem affecting all population groups, but especially pregnant women and young children [1]. For pregnant women, the consequences of anaemia include mortality, poor pregnancy and birth outcomes including premature delivery, low birth weight and increased perinatal mortality [1–3]. The most significant contributor to the onset of anaemia is iron deficiency [1]. The World Health Organisation (WHO) estimates that iron deficiency anaemia (IDA) affects almost half of the world’s pregnant women and pre-school children with a prevalence of over 65 % in Africa and Asia, and that it causes (directly or indirectly) one fourth of all maternal deaths [3]. In The Gambia, iron deficiency anaemia among women and children has been found to be high and of public health significance with 73 % of pregnant women and 56 % of lactating women being anaemic [4].

Although iron deficiency with or without anaemia has important consequences for human health and child development, there has been an absence of international agreement on how best to assess the iron status of populations. Serum ferritin (SF) is one of the few biochemical indices of which low levels reflect depleted iron stores [5, 6] but it is known to be raised by infection and inflammation as it is an acute phase protein and thus has very high false negative rates in least developed countries [7]. Similar problems also arise with the other commonly used iron status indicators as summarised in Table 1 below.

**Table 1:** Limitation of current methods of assessing IDA

Test	Measure	Limitation
Stain bone marrow preparation	Iron stores	Expensive, invasive and traumatic
Haemoglobin (Hb)	Anaemia	Does not measure ID per-se
Serum ferritin (SF)	Iron stores	Raised by infection and inflammation
Zinc protopopherin (ZnPP)	Iron in new cells	Affected by infection and inflammation
Soluble transferrin receptor (sTfR)	Severe ID even with inflammation	Affected by (>) red cell prod. Lack standardised reference for measure’
sTfR/logSF ratio	Iron stores	Lack standardised assay range. Ferritin affected by infection or inflammation
Transferrin saturation (TSAT)	Iron levels	Affected by (>) plasma concentration
Serum iron	Iron in sera	Affected by recent iron ingestion and infection
Total iron binding capacity (TIBC)	Iron in serum	Affected by infection

Hepcidin is a peptide hormone that has been shown recently to be the master regulator of iron absorption and distribution in humans [8–11]. The potential for hepcidin as a superior marker for iron deficiency has been highlighted in many recent studies [12–16]. Hepcidin controls iron homeostasis by inhibiting dietary iron absorption, release of iron in the macrophages and reducing iron flow to the erythron [8, 11, 17–19]. This it does by binding to the iron exporter ferroportin inducing its internalisation and degradation [18].

Recent studies have noted that over the course of a malaria season, hepcidin integrates signals arising from parasitaemia, inflammation and anaemia [7, 20]. The fact that hepcidin plays a crucial role in the above signals and acts both as a reporter of iron status and a regulator of iron absorption, distribution and metabolism suggests it may be the ideal index for iron deficiency and could form the basis of a PoC diagnostic for iron deficiency in at-risk population groups in developing countries [20].

WHO, originally recommended universal iron supplementation for all pregnant women with a dose of 60 mg iron and 400ug folic acid daily [21]. However, a recent study has shown that pregnant women who are anaemic and iron deficient may be protected from malaria [22]. A recent review also indicate that pregnant women who received daily iron and folic acid supplementation are at a greater risk of haemoconcentration (haemoglobin greater than 130 g/L) in the second and third trimester of pregnancy [23]. Although the effect of the haemoconcentration in the above review was uncertain, Ziaei et al. [24] in a randomised controlled trial (RCT) of over 700 pregnant women who took 50 mg iron as ferrous sulphate daily found that small-for-gestational-age birth rate and the number of women with hypertensive disorders increased significantly. They concluded that routine iron supplementation in non-anaemic women is not rational and may be harmful. Recently, two hazardous complications of pregnancy; gestational diabetes mellitus and preeclampsia have been recognized to be associated with elevated body iron levels [25].

There has been little specific evidence on the relationship between risk of malaria and other infections with iron status and iron supplementation in pregnant women. Yet, the benefits of iron supplementation must be carefully weighed against the risks in developing countries [26, 27].

There is now some evidence that smaller doses of 30 mg iron daily could achieve similar results as the daily 60 mg iron [21]. A Cochrane review on the treatments of iron deficiency anaemia in pregnancy [28] indicated that daily low dose iron supplementation may be effective at treating anaemia in pregnancy with fewer gastrointestinal side effects compared with higher doses. WHO has now recommended the use of doses between 30 and 60 mg for daily supplementation for pregnant women [29]. Further evidence suggest that the use of multiple micronutrient supplements with three or more micronutrients is associated with a 39 % reduction in maternal anaemia compared with placebo or with two micronutrients or fewer (relative risk 0 · 61, 95 % CI 0 · 52—0 · 71). Multiple micronutrient supplementation is also known to result in a decrease in the risk of low-birth weight babies (0 · 83, 0 · 76—0 · 91) and small-for-gestational-age babies (0 · 92, 0 · 86—0 · 99) [30].

In this proof of concept study we aim to test the hypothesis that a screen-and-treat approach to iron supplementation will achieve similar efficacy in combating ID and IDA at a lower overall dosage of iron. The assumption that lower doses will improve safety and tolerability will also be tested. The design will establish whether using screen-and-treat with UNIMMAP containing either 60 mg or 30 mg iron per day is non-inferior to UNIMMAP containing 60 mg/day as a universal daily supplement.

## Design/Methods

### Study design

This study is designed as a proof-of-concept, 3-arm, double blind, RCT over a period of 12 weeks with a sample of 462 pregnant women randomly assigned to receive: a) UNIMMAP containing 60 mg/day iron; b) UNIMMAP containing 60 mg/day iron but based on a weekly hepcidin screening indicating if iron can be given for the next 7 days or not; c) UNIMMAP containing 30 mg/day iron based on screening as in (b).

### Determining the hecidin cut-off value

The hepcidin cut-off of <2.5 ng/mL as a threshold (to receive iron or not) is based on the analysis of sera from 270 pregnant women participating in the ENID study [31] with samples available for 3 time points (12–14 weeks, 20 weeks and 30 weeks gestation). A receiver operating characteristics (ROC)-curve was generated to measure the area under the curve (AUC^ROC^). Method described elsewhere [12].

### Study location and participants

The Hepcidin and Anaemia in Pregnancy (HAPn) study will be carried out in 12 communities of Jarra West and Kiang East (rural Gambia) about 150 km from the capital city of Banjul. The Regional Health Team, the health facilities within the study area and the individual communities have been sensitised and their approval gained for conducting the study.

The study will involve 462 healthy pregnant women between the ages of 18 and 45 years (established by asking, use of birth certificates, identity cards or calendar events) who are pregnant (estimated at between 14 and 22 weeks gestation, by fundal height assessment and date of last menstrual period (LMP)) and are likely to be resident in the study area for the duration of the study period.

Pregnant women who are identified as potential participants will be excluded from the study if found to be (i) severely anaemic (<7 g/dL), (ii) seriously ill (infectious disease of clinical significance) at recruitment (iii) suffer from a chronic disease (iv) have pregnancy complications (e.g., pre-eclampsia) at enrolment or (v) already participating in another study.

## Detailed study procedure

### Screening and enrollment (Baseline)

Pregnant women living within the two health facility catchment areas will be identified as they visit the Reproductive and Child Health (RCH) clinics to register and book their pregnancies. As part of the routine services provided, a nurse midwife determines their stage of pregnancy. If a woman is within the window of the study (14–22 weeks gestation), she will be invited by the study team to take part in the study and informed consent will be sought. Once a signed informed consent is obtained and all of the inclusion and none of the exclusion criteria are met, she will be enrolled in the study, and asked to provide 5 mL venous blood (Day 0 below). Participants will thereafter be assigned to one of the 3 study groups (see randomisation, below). All women enrolled in to the study will be provided with long lasting insecticide-treated bed nets (LLINs).

### Follow-up

Following recruitment, women will be followed up weekly in their communities. Each week, trained MRC field assistants (FA) will invite the study participants to a central location within their communities for collection of a finger prick blood sample for analysis of haemoglobin (Hb) using a HemoCue Hb 301 analyser (HemoCueAB, Angelholm, Sweden), malaria parasitaemia using a SD Bioline One step malaria antigen *Pf* Test (SD Standard Diagnostics, Inc. Kyonggi-do, Korea) and hepcidin levels using the BACHEM Hepcidin-25 ELISA. Hb and malaria assessments will be performed immediately; samples for hepcidin measurements will be transferred on ice to a laboratory at MRC Keneba where analysis will commence within the hour of arrival. The following day hepcidin results will be available and a 7 day supply of supplements packed according to the hepcidin results (computer generated). The day after, participants will be provided with their supplements. While the supplements are being distributed, the FA will also assess beneficial effects, adverse events and compliance. All activities will be documented on a case report form (CRF) using electronic data capture in the form of a hand held device (SAMSUNG Galaxy Tab3 Model SM-T211). Data will be sent through a secure internet connection to the MRC database.

### Ethics and safety monitoring

The trial has been approved by the Medical Research Council (MRC) Scientific Coordinating Committee (SCC) and the Joint Gambia Government MRC Ethics Committee. It will be overseen by a Data Safety Monitoring Board (DSMB) and a Trial Steering Committee assisted by a Trial Monitor (TM). Together they will be responsible for reviewing all interim data, treatment safety and efficacy including the protection of the rights and wellbeing of the participants. The trial will be conducted according to Good Clinical Practice (GCP) principles taking in to consideration the provisions of the World Medical Association (WMA) Declaration of Helsinki (October 2013).

Participants will be monitored on each scheduled follow up day for all adverse events (AEs) defined as any untoward or unfavourable medical occurrence in a human subject, including signs and symptoms which are temporally associated with the research procedure or trial intervention, whether or not considered related to the subject’s participation in the research. All serious adverse events (SAEs) defined as any AE that is life-threatening or results in death or require hospitalisation or prolongation of hospitalisation, is a persistent or significant disability/incapacity or is a congenital anomaly/birth defect or a reported maternal death, miscarriage or stillbirth will be recorded as SAEs and investigated by a physician. Monitoring of the participants will then continue until they deliver and the outcome of the pregnancy for both mother and child is known (postnatal check-up within 72 h after delivery).

## Collection and analyses of biological samples during enrollment and follow-up visits

As described, finger prick blood samples will be collected weekly. Additional 5 mL venous blood samples will also be collected at 4 different time-points (Days 0, 14, 49 and 84) within the 12 week period of the study. As intermittent preventative treatment (IPT) is routine in this region, participants will receive their IPT dose immediately after blood draws are done at days 14 and 49 in order not to influence our *ex vivo P. falciparum* assays. All venous blood draws will be carried out by the study nurse and finger prick blood samples by the field assistants (FAs).

Full haematology including haemoglobin and reticulocytes will be assessed on samples collected on Days 0, 14, 49 and 84 in a 1.2 mL EDTA Sarstedt tube using the Medonic M Series analyser.

Biochemistry analysis of plasma ferritin, iron, transferrin saturation (TSAT), soluble transferrin receptor (sTfR), C-reactive protein (CRP), and alpha-1-acid glycoprotein (AGP) will be measured by Cobas Integra 400+ using 500 μl from −20 frozen samples. The Cobas measuring principle for ferritin, transferrin and CRP will be via turbidimetric principle at 552 nm, 340 nm and 552 nm respectively and for iron, FerroZine method without deproteinization. sTfR will be measured photometrically at 583 nm and alpha-1-acid glycoprotein will be through turbidimetric. Hb genotyping will be performed using Hb electrophoresis with Shandon Vokam 400 on all samples collected on Day 0.

This study is not powered to use clinical endpoints to assess safety. Instead the trial will use in vitro assays to assess safety on each of the four venous samples per subject. *Ex vivo* growth of *P. falciparum* will be assessed in washed red blood cells (RBCs) using a field-ready 96-well plate method with florescence-activated cell sorting (FACS) readout [32]. A small subset of RBCs will be lysed for measurement of riboflavin status by the erythrocyte glutathione reductase activation coefficient (EGRAC) test because this may affect RBC stability.

The *ex vivo* growth of sentinel organisms (*Escherichia coli, Yersinia enterocolitica, S. enterica* serovar (Typhimurium), *Staphylococcus epidermidis, Staphylococcus aureus and Candida albicans)* analyses will be performed in frozen (−20 °C) plasma (400 μl) as previously described in the investigation of the effects of iron supplementation on pathogen virulence in human serum [33].

DNA will be extracted from baseline whole blood samples to study the genes implicated in iron metabolism. Known genetic risk factors for malaria assays will include alpha-thalassemia, G6PD and sickle traits. Furthermore, putative functional and key tagging variants in iron regulatory and inflammatory pathways will be screened.

## Randomisation and blinding

### Randomisation

Recruited women will be randomly assigned (computer generated) to one of the 3 treatment arms (equal number in each arm) balanced by the Hb concentration of the baseline blood sample and gestational age. To achieve this, at each day of recruitment, subjects will be categorised into two Hb classes (above and below the median Hb of the respective day) and according to 2 gestational age periods (14–18 weeks, 19–22 weeks) making 4 classes. In each of the 4 classes, the women will be randomly assigned to the 3 treatment arms using a predetermined block randomisation.

The randomisation database of treatment arms (A, B, C) will be password protected with the database developer and his assistant knowing the password. If a subject needs to be unblinded at the request of the DSMB, their treatment can be easily identified without unblinding the whole study.

### Blinding

**Fig. 1:**
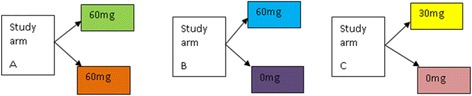
An example of blinding using colour codes

Participants, field workers, study nurse and PI will be blinded as to which treatment group participants belong to and which supplement participants receive each week. The supplements will be pre-packed on a weekly basis by the field coordinator in Keneba using lists automatically (computer) generated by the data office taking into account the hepcidin results of the participants. The list will indicate the letter of the supplement the participant receives for the following 7 days but the field coordinator will not know which code is allocated to which supplement. The capsules are coded (2 codes for treatment arm A (60 mg and 60 mg iron), 2 codes for arm B (60 mg and 0 mg iron) and 2 codes for arm C (30 mg and 0 mg iron), see Fig. 1. The pre-packed weekly supplies labelled with each participant’s ID will be handed over to the PI who will be responsible for handing them over to the field workers who will distribute to the individual participants. The laboratory staff and data entry clerks will also be blinded.

The allocation of the colour code will be done by 2 people independent of the study and the key will be kept in a locked cabinet in Keneba. The blinding for the study may be broken if in any of the 3 treatment arms, safety issues arise and the trial team is advised by the DSMB to do so.

## Investigational product

The investigational product to be used is the UNICEF/WHO/UNU international multiple micronutrient preparation (UNIMMAP). Three products will be administered (UNIMMAP with 60 mg iron, UNIMMAP with 30 mg iron, UNIMMAP with 0 mg iron). All formulations also contain 400 ug folic acid and 14 other micronutrients (Table 2). The UNIMMAP supplement has already been used safely in other pregnancy trials [34]. The formulations are produced by DSM South Africa under Good Manufacturing Practice (GMP) conditions where it will also be dosed into gelatin capsules and packed in tubs. The labelling will include a statement that ‘trial medications are only for use of trial participant’.

**Table 2:** Intervention product - Formulation based on UNU/UNICEF/WHO supplement called UNIMMAP

Micronutrients	Dose/day
Vitamin A (ug RE)	800
Vitamin D (IU)	200
Vitamin E (mg)	10
Thiamine (mg)	1.4
Riboflavin (mg)	1.4
Niacin (mg)	18
Folic acid (ug)	400
Vitamin B6 (mg)	1.9
Vitamin B12 (ug)	2.6
Vitamin C (mg)	70
Zinc (mg)	15
Iron (mg)	60 or 30 or 0 (placebo)
Iodine (ug)	150
Selenium (ug)	65
Copper (mg)	2

The products will be stored under controlled conditions (in an air-conditioned storage at around 20 °C) at MRC Keneba. The product is stable for 18 months if kept under these conditions.

Each participant will receive 1 capsule per day. Each week field workers will be visiting study participants to distribute the respective weekly supply (7 capsules) to each study participant. The participants will be instructed to take 1 capsule a day with water or another drink. Each time the field workers distribute the new weekly supply of capsules they will account for the number of capsules consumed/not consumed from the previous week in order to check for compliance. Any left-over capsules will be collected by the field workers.

## Sample size and statistical analysis plan

### Sample size determination

Using the haemoglobin data obtained from pregnant women enrolled in the ENID study in West Kiang [31], a SD of 1.28 was derived. This was used to obtain a sample size of 154 participants for each of the 3 arms (Table 3) calculated using a 1-sided α (alpha) of 2.5 with a conservative-Bonferroni type correction (÷3) so as not to inflate the type 1 error rates while performing multiple tests. A total sample size of 462 pregnant women followed up for 12 weeks with a less than 10 % loss to follow-up will provide 80 % power to establish that:

**Table 3:** Study arms

Group	Dose (mg/day Fe)	Universal (N)	Screen with Hepcidin (Yes or No)
A	60	154	No
B^a^	60	154	Yes
C^a^	30	154	Yes

^a^Groups B and C will be tested weekly and only given their next seven day supply of iron if plasma hepcidin falls below cut-off for ‘safe and ready’

1. arm B is non-inferior to arm A on the primary endpoint defined below.
2. arm C is non-inferior to arm A at the same level as described in the statistical analysis plan.
3. arm C is non-inferior to arm B.

Note: it is being stated *a priori* that arm C will be compared with B for non-inferiority to explore if the results can influence policy on the further lowering of the dose of iron for those not iron deficient. The study (sample size) is powered for this analysis.

### Statistical analysis plan

The approach to the analysis for this trial will be a test of non-inferiority. As recommended for the acceptance of non-inferiority analysis, a per-protocol (PP) analysis will be performed. Additional analysis including all Hb measurements (not only day 84) will be explored. These will be described in a more detail statistical analysis plan.

## Primary endpoint

**Fig. 2:**
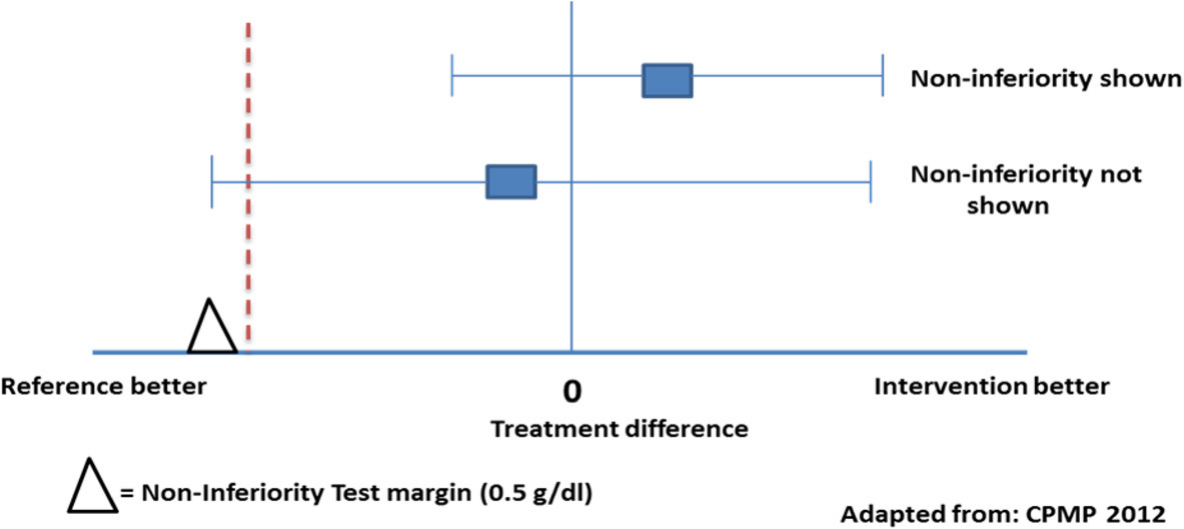
Confidence interval approach to analysis of non-inferiority trial

The primary non-inferiority endpoint is pregnancy-adjusted haemoglobin at Day 84. To adjust for multiple testing (3 arms), non-inferiority will be tested with a 96.7 % CI of the lower 0.83 % (2.5 %/3) limit for the difference. The lower confidence limit for the difference in haemoglobin concentration between the universal and screened treatments on Day 84 will be above −0.5 g/dL ( −5 g/L), the smallest value considered to be of minimum public health relevance. See illustration in Fig. 2.

## Secondary endpoints

i. Proportion of Hb < 11 g/dL (%) at Day 84.
ii. Hepcidin at Day 84 (as a continuous variable but also using a cut-off point of >2.5 ng/mL to calculate the proportion).
iii. sTfR/log-ferritin ratio (ferritin index <2.0) at Day 84 (continuous variable but a proportion will also be calculated).
iv. Iron deficiency anaemia (IDA) defined as Hb < 11 g/dL and ferritin < 15 ug/L when CRP is < 5 mg/L OR Hb < 11 g/dL and ferritin < 30 ug/L when CRP is > 5 mg/L and ferritin index < 2.0.
v. Iron dosage (number of weeks supplemented).
vi. Adverse events that may include nausea/vomiting, dizziness, constipation, black stool, stomach discomfort assessed weekly will be evaluated at Day 28 and Day 84. In addition, an aggregate score will also be assessed.
vii. Compliance.
viii. *P. falciparum* growth in serum (difference between in vitro growth rates at Days 0, 14, 49 and 84).
ix. *Ex vivo* growth of sentinel bacteria (difference between in vitro growth rates at Days 0, 14, 49 and 84).

Analysis of the continuous variables (ii) and (iii) will be based on comparing means using a t-distribution where 95 % CI is calculated for the difference in arm A compared to B and C respectively and also between B and C. A logarithmic transformation will be applied to non-normally distributed variables and unpaired t-tests done on the transformed data. For variables (i), (iv), (v), (vi), (vii), (viii) and (ix), a frequency distribution using *X*^2^ comparing proportions between the arms will be performed with a statistical test for significance for the difference between the arms set at 5 % (*P* < 0.05). Additionally, adverse events will be analysed using multiple regression analysis controlling for possible confounders to see which events or aggregate score are associated to which arm. Compliance will be assessed comparing proportions consumed in each arm with number consumed as numerator and the total number of capsules prescribed between enrolment and end of study as the denominator. STATA 12.1 or any of its latest versions will be used for all the analyses.

Further exploratory analysis will be conducted and the endpoints include:

Primary endpoints adjusted for CRP, AGP and malaria (we decided *a priori* that the primary endpoints will not be adjusted for the above, however, we wish as part of an exploratory analysis to adjust for them), sTfR, Ferritin, and TSAT.

## Informed consent and confidentiality

All field workers taking part in the recruitment of participants will be trained on translating and issuing of the informed consent documentation. The information sheet will be translated to all the illiterate participants in a language that they understand in the presence of an independent witness. The literate participants will be allowed to read the information sheet in their own time. Participants will also be encouraged to ask questions and seek clarification from the field workers and the PI. If the participant agrees to take part in the study, informed consent is recorded by a signature or thumbprint.

Participants will be allocated an individual identification number. Participant identification numbers will be used on all samples and data forms generated during the course of the study. Following sample collection and data entry, linkage of the ID back to the study participant will not be possible without a lookup table, which will be held by the data manager only and his designated data staff during the course of the study. The field worker will have a printout version of the list. Once data collection is complete, analysis will be performed on an anonymised copy of the data. All forms and case report forms (CRF) will be kept in locked files. At all stages, staff/collaborators responsible for sample analysis will be blinded as to the subject’s identification. Together, these processes will ensure complete confidentiality of the data gathered and impartiality of data analyses.

## Discussion

WHO has identified the need for a lower dose in iron supplementation as recommendations being used by countries can pose risks to some pregnant women. This trial will test the efficacy of employing a screen-and-treat approach to minimise iron exposure whilst achieving a similar therapeutic effect.

As 50 % of anaemia in pregnancy is assumed to be due to ID, the assessment of iron status (not only anaemia) in supplementation programmes is critical and hepcidin has shown the potential of being an improved bio-marker for iron status and therefore a signal for the safe administration of iron in pregnancy. In this study we will explore this potential of hepcidin with a pre-determined cut-off value of <2.5 ng/ml to screen for the readiness to receive iron. When iron is needed, we will supplement using oral, tablet form UNIMMAP formulation containing 60 mg iron daily (universal) on one hand or provide 60 or 30 mg iron daily only when hepcidin levels are below the threshold cut-off value mentioned above (screen-and-treat). We hypothesise that a screen-and-treat approach to iron supplementation will achieve similar efficacy in combating ID and IDA at a lower overall dosage of iron as therapy will be targeted to periods when the enterocyte iron absorption channels are open. We assume that lower doses will improve safety and tolerability and these will be tested as secondary outcomes.

In summary, this trial will contribute towards minimising exposure to excessive iron that may not be needed and may indeed be harmful and allow the pregnant woman to maximise the absorption and utilisation of iron when it is most needed. In addition, the overall lower dosage may help decrease side-effects and increase compliance. The exploration of hepcidin as a potential for a simple PoC diagnostic to screen for the readiness to receive iron will assist health workers to make the right decisions for iron supplementation which will help in improving health care delivery and reduce maternal morbidity and mortality as part of efforts to meet the Millennium Development Goal 5 and the forthcoming Sustainable Health Agenda.

## Abbreviations

AGP, alpha-1 acid glycoprotein; CRF, case report form; CRP, C-reactive protein; DSM, Dutch State Mines; ELISA, enzyme-linked immunosorbent assay; FA, field assistant; FACS, florescence-activated cell sorting; FW, field worker; GMP, good manufacturing practice; Hb, haemoglobin; ID, iron deficiency; IDA, iron deficiency anaemia; LLINs, long lasting insecticide-treated nets; MRC- ING, Medical Research Council International Nutrition Group; MRC, Medical Research Council; represents Medical Research Council Unit, The Gambia; PoC, point of care; RCH, reproductive and child health; RCT, andomised control trial; RDT, rapid diagnostic test; SAT, transferrin saturation; SCC, Scientific Coordinating Committee; SF, serum ferritin; sTfR, soluble transferrin receptors; TM, trial monitor; UNIMMAP, UNICEF/WHO/UNU International Multiple Micronutrient Preparation; WHO, World Health Organisation; WIMM, Weatherall Insttitute of Molecular Medicine 
